# A Key Hydrophobic Patch Identified in an AAA^+^ Protein Essential for Its *In Trans* Inhibitory Regulation

**DOI:** 10.1016/j.jmb.2013.04.024

**Published:** 2013-08-09

**Authors:** Nan Zhang, Timothy Simpson, Edward Lawton, Povilas Uzdavinys, Nicolas Joly, Patricia Burrows, Martin Buck

**Affiliations:** 1 Division of Cell and Molecular Biology, Sir Alexander Fleming Building, Imperial College London, Exhibition Road, London SW7 2AZ, UK; 2 Institut Jacques Monod, CNRS UMR 7592, Université Paris Diderot, Batiment Buffon, 15 rue Helene Brion, 75205 Paris cedex 13, France

**Keywords:** bEBP, bacterial enhancer binding protein, AAA^+^, ATPases Associated with various cellular Activities, PspA, phage shock protein A, PspF, phage shock protein F, RNAP, RNA polymerase, WT, wild type, PDB, Protein Data Bank, EDTA, ethylenediaminetetraacetic acid, AAA^+^ proteins, PspF, PspA, enhancer binding protein, σ54

## Abstract

Bacterial enhancer binding proteins (bEBPs) are a subclass of the AAA^+^ (ATPases Associated with various cellular Activities) protein family. They are responsible for σ^54^-dependent transcription activation during infection and function under many stressful growth conditions. The majority of bEBPs are regulated in their formation of ring-shaped hexameric self-assemblies via an amino-terminal domain through its phosphorylation or ligand binding. In contrast, the *Escherichia coli* phage shock protein F (PspF) is negatively regulated *in trans* by phage shock protein A (PspA). Up to six PspA subunits suppress PspF hexamer action. Here, we present biochemical evidence that PspA engages across the side of a PspF hexameric ring. We identify three key binding determinants located in a surface-exposed ‘W56 loop’ of PspF, which form a tightly packed hydrophobic cluster, the ‘YLW’ patch. We demonstrate the profound impact of the PspF W56 loop residues on ATP hydrolysis, the σ^54^ binding loop 1, and the self-association interface. We infer from single-chain studies that for complete PspF inhibition to occur, more than three PspA subunits need to bind a PspF hexamer with at least two binding to adjacent PspF subunits. By structural modelling, we propose that PspA binds to PspF via its first two helical domains. After PspF binding-induced conformational changes, PspA may then share structural similarities with a bEBP regulatory domain.

## Highlights

• What is the mechanism of *in trans* inhibition of oligomeric self-assemblies?
• Inhibitor initially docks on the AAA^+^ domain at a hydrophobic patch.
• Consequently, ATPase and self-association of the AAA^+^ domain are altered.
• Inhibitor’s structure mimics the evolutionarily divergent *in cis* regulatory domain.
• *In trans* inhibition of oligomeric AAA^+^ domains requires multiple contacts.

## Graphical Abstract

**Figure:**
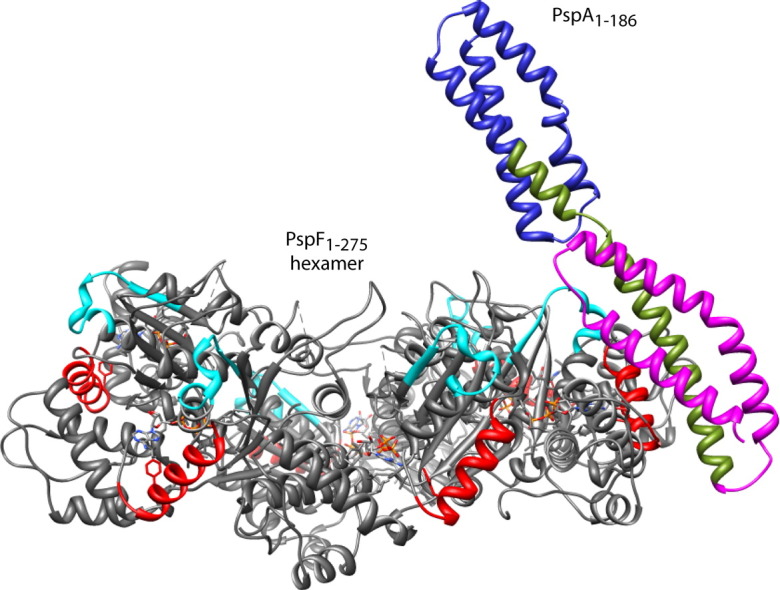

## Introduction

**Fig. 1:**
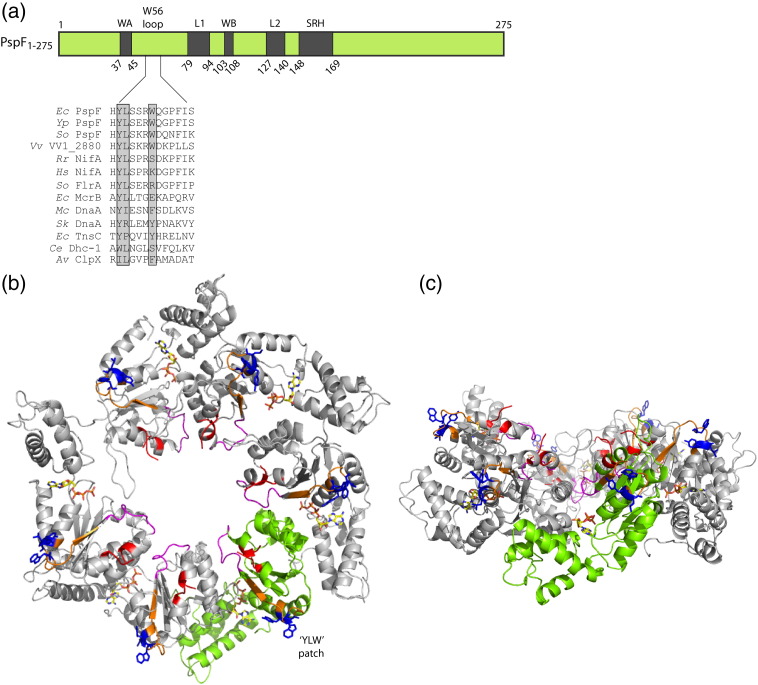
Domain organisation and structure of PspF_1–275_. (a) The AAA^+^ core of PspF (PspF_1–275_) contains the functional motifs required for self-assembly, ATPase, and transcription activation: walker A (WA) for ATP binding, loop 1 (L1) for σ^54^ and DNA binding, walker B (WB) for ATP hydrolysis, loop 2 (L2) for L1 coordination, and second region of homology (SRH) that contains the *trans*-acting arginine fingers. The surface-exposed W56 loop (residues 50–62) proposed to be vital for PspA inhibition is aligned against AAA^+^ homologues from the following organisms: *E*. *coli* (*Ec*), *Yersinia pestis* (*Yp*), *Shewanella oneidensis* (*So*), *Vibrio vulnificus* (*Vv*), *Rhodospirillum rubrum* (*Rr*), *Herbaspirillum seropedicae* (*Hs*), *Mycoplasma capricolum* subsp. *capricolum ATCC 27343* (*Mc*), *Synechocystis* sp. *PCC 6803* substr. *Kazusa* (*Sk*), *Caenorhabditis elegans* (*Ce*), and *Azotobacter vinelandii* (*Av*). A very hydrophobic patch within the W56 loop was found in many clades of the AAA^+^ proteins (highlighted in grey). (b and c) Two views of the PspF_1–275_ hexamer modelled by Rappas with ATP-bound monomers (PDB entry 2C96) based on energy minimisation.^5,6^ The PspF_1–275_ hexamer was depicted in grey ribbons with one protomer highlighted in green to delineate the boundaries of the self-association interface. Key residues (the YLW patch, blue) in the W56 loop (orange), L1 (red), linker 1 (magenta), and ATP (rainbow) were highlighted in the hexameric structure.

The AAA^+^ (ATPases Associated with various cellular Activities) protein family is a large group of macromolecular assemblies that are present in all kingdoms of life. They perform essential cellular functions ranging from DNA replication, transcription, microtubule trafficking, to protein homeostasis.^1–3^ AAA^+^ protein dysfunction has been linked to a number of neurological and motor-degenerative diseases in humans (reviewed in Ref. 2). Recently, a Clade 3 AAA^+^ protein has been associated with vesicle formation and co-localisation of viral RNA replication complexes in the foot-and-mouth disease virus.^4^ In order for AAA^+^ proteins to engage and induce conformational changes in a diverse array of substrates, they must form higher-order oligomers (often hexamers, sometimes heptamers). After receiving signals from the *cis*- or *trans*-acting regulatory domains, the ubiquitous AAA^+^ core (typically 200–275 amino acids long, comprising all key functional motifs, Fig. 1a) converts chemical energy into mechanical motion at the expense of nucleotide hydrolysis.

Bacterial enhancer binding proteins (bEBPs) belong to Clade 6 of the AAA^+^ protein family and are activators of the σ^54^-dependent transcription paradigm.^1^ σ^54^ directs the RNA polymerase (RNAP) to promoters specific to changes of environmental cues, such as nitrogen assimilation, phage shock, pathogenicity, biofilm formation, and bioluminescence (Refs. 7–9 and reviewed in Refs. 10 and 11). σ^54^ binds directly to the ‘-24’ GG promoter element and, by imposing a network of inhibitory interactions around the ‘-12’ GC promoter element, contributes to a high-energy barrier, preventing DNA opening.^12–15^ Consequently, spontaneous isomerisation from the closed complex (RP_C_) to the open complex (RP_O_) is impeded. Hexameric bEBPs overthrow these inhibitory interactions at the ‘-12’ site by relaying the ATP hydrolysis energy (via σ^54^ Region I and promoter DNA) to RP_C_ reorganisation, leading to full DNA melting.^5^

The well-characterised *Escherichia coli* phage shock protein F (PspF), unlike most bEBPs, does not contain a *cis*-acting regulatory domain. Instead, its activity is negatively regulated *in trans* by phage shock protein A (PspA) in order to respond to inner membrane stress.^10,16–18^ Analogous to the homologue Vipp1 *in planta*, PspA is composed of four α-helical domains.^19^ Deletion of the last α-helical domain generates the PspA_1–186_ variant that is as active as the full-length PspA in PspF binding and inhibition *in vitro*.^20^ The ratio of PspA to PspF in the PspA–F inhibitory complex is approximately 1:1 (i.e., six PspAs per PspF hexamer) and inhibition is probably exerted via the PspF residue W56 located in a surface-exposed loop (Fig. 1b and c^20^). Under membrane stress conditions, for example, the membrane disruption by filamentous phage pIV proteins, PspA dissociates from the inhibitory complex to form large 36mers, releasing PspF for transcription activation. These 36mer PspA rings show a 9-fold symmetry, have a dimension of 200 Å (outer diameter) × 85 Å (height), and have a calculated mass of 1034 kDa.^21^ Such large PspA assemblies are proposed to bind directly to liposomes, thereby suppressing proton leakage.^22^

Defining a contact surface in PspF responsible for PspA binding is important for establishing the mechanism of negative regulation. Guided by bioinformatics, Elderkin *et al.* identified within the AAA^+^ core of PspF (PspF_1–275_) a variant (W56A) that can bypass PspA negative regulation on ATP hydrolysis, σ^54^-DNA isomerisation, and *in vivo* transcription activation by diminishing PspA binding.^20,23^ The authors proposed that residue W56 (possibly along with other surface-exposed residues in proximity) directly constituted the PspA binding site.^20^ Many of these surface-exposed residues are located in a flexible loop that connects the C-terminus of helix 2 and the N-terminus of β-sheet 2 in PspF_1–275_. For convenience, this entire region (residues 50–62) is collectively called the ‘W56 loop’ in this study. Potentially, the intramolecular residues that support the stability of the W56 loop may account for signal transduction from the PspA binding site to the ATP hydrolysis site. By systematically substituting individual W56 loop residues with Cys, we demonstrated their strong functional association with the ATP hydrolytic site and the PspF self-association interface. We identified a hydrophobic patch composed of a Tyr, a Leu, and a Trp within the W56 loop. Site-specific UV cross-linking data suggest that this ‘YLW’ patch should be the primary docking site for PspA. By computational analyses, we were able to obtain a PspA_1–186_ tertiary structure. We propose that the PspA_1–186_ may functionally resemble a *cis*-acting regulatory domain and undergo conformational changes at the outer rim of the bEBP hexamer upon initial docking.

## Results

### Substitutions in the PspF W56 loop affect transcription activation and three of its Cys variants escape the negative regulation of PspA_1–186_

One proposed PspA-interacting residue in PspF, W56, is located in a loop (named W56 loop) on the surface of the AAA^+^ hexameric plane (Fig. 1b and c). The solvent-exposed W56 loop may be directly available for interaction with PspAs. We mutated each position of the W56 loop with a Cys residue, generating 13 PspF_1–275_ Cys variants for subsequent functional and conjugation studies (such as FeBABE and FRET).

**Fig. 2:**
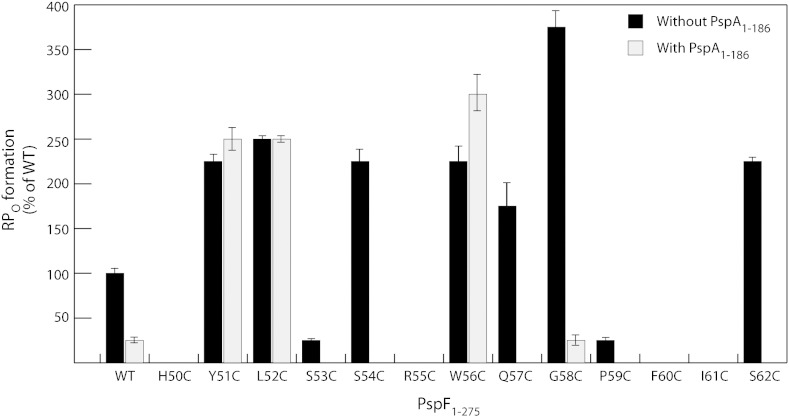
RP_O_ formation assay of the W56 loop variants in the presence and absence of PspA_1–186_. RP_O_ generated from a supercoiled *S. meliloti nifH* promoter was directly correlated with 5′-UpG dinucleotide-primed transcript UpGpGpG.^24^ The amount of RP_O_ formed with each variant was expressed as a percentage of that of PspF_1–275_ WT in the absence of PspA_1–186_.

To investigate whether the Cys incorporation affected transcription activation and PspA-dependent inhibition of PspF_1–275_, we performed an *in vitro* open promoter complex (RP_O_) formation assay. Each Cys variant was mixed with the σ^54^-RNAP holoenzyme, dATP for AAA^+^ domain hydrolysis, and supercoiled *Sinorhizobium meliloti nifH* promoter DNA. The amount of − 1, + 1 dinucleotide-primed transcript (UpGpGpG) generated reflects directly the amount of RP_O_. As shown in Fig. 2, Cys incorporation in the W56 loop resulted in three RP_O_-related phenotypes in the absence of PspA_1–186_ (black bars): (i) better than wild-type (WT) transcription activation (Y51C, L52C, S54C, W56C, Q57C, G58C, and S62C), (ii) significantly reduced transcription activation (S53C and P59C), and (iii) complete loss of transcription activation (H50C, R55C, F60C, and I61C). When the Cys variants were pre-incubated with PspA_1–186_ (recall that PspA_1–186_ is as effective as full-length PspA in PspF inhibition), nearly all RP_O_ formation was reduced by at least 3-fold (Fig. 2). Interestingly, variants Y51C, L52C, and W56C were able to escape this inhibition (Fig. 2). A direct protein binding assay revealed that both Y51C and L52C failed to stably engage PspA_1–186_ (Table 1 and Supplementary Fig. 1), thereby explaining their insensitivity to PspA_1–186_ negative regulation. In contrast, the W56C variant still appears able to bind PspA_1–186_ weakly (Table 1 and Supplementary Fig. 1). The fact that W56C can escape PspA_1–186_ negative regulation suggests that either an intramolecular pathway for activation must be re-routed or levels of PspA binding are insufficient for inhibition. Taken together, we have shown that the W56 loop contains critical residues for RP_O_ formation. We also successfully identified three W56 loop variants (Y51C, L52C, and W56C) that can bypass PspA inhibition. The three residues may form a hydrophobic patch (the YLW patch) for PspA engagement.

### Variation in the W56 loop has a detrimental impact on ATP hydrolysis

In σ^54^-dependent transcription, the isomerisation process from RP_C_ to RP_O_ is rarely spontaneous and requires additional energy inputs derived from ATP binding and hydrolysis. This energy must be coupled via an intramolecular route from the self-association interface of PspF where ATP is bound and hydrolysed to the surface-exposed loop 1 (L1), which touches the RP_C_, and subsequently to the mechanical motions that disrupt the RP_C_.

Potentially, the W56 loop supports or impacts upon the intramolecular energy coupling route to L1 and may communicate with the ATP hydrolysis site. As a result, the Cys mutations of the W56 loop could affect ATP hydrolysis, leading to a deregulated PspA-insensitive RP_O_ formation as we observed for some substitutions within the intramolecular signalling pathway.^25^ To test this proposal, we performed ATPase assays under ATP saturating conditions. The Cys substitutions tested all caused a significant reduction in ATP hydrolysis, with the least detrimental effect being a near 50% reduction (Table 1). Such observations suggest a strong functional linkage between the W56 loop and the ATP hydrolytic site, which is created between two adjacent subunits of the hexameric ring. Recall that disruption of this proposed functional linkage has been shown to decouple PspA binding from the inhibitory effect in a number of PspF N64 variants.^25^ Interestingly, several of the Cys variants were able to effectively activate transcription with extremely reduced ATPase activities: Q57C (175% of the WT RP_O_ activity and 8% of the WT ATPase activity), G58C (375% of the WT RP_O_ activity and 5% of the WT ATPase activity), and S62C (225% of the WT RP_O_ activity and 2% of the WT ATPase activity). This observation suggests that a WT ATP turnover rate of PspF is not an optimum for efficient transcription activation and that the efficiency of energy coupling reaction between RP_C_ and PspF_1–275_ can be modulated through the sequence of the W56 loop and, by inference, binding interactions made by PspA with this loop.

### W56 loop plays a modest role in stable engagement of σ^54^

The isomerisation from RP_C_ to RP_O_ requires direct contacts between PspF and σ^54^ for the energy coupling step.^5,24,26^ However, such interaction is transient and can be difficult to capture throughout the ATP binding and hydrolysis cycle. We have circumvented this problem by using nucleotide analogues to ‘trap’ the co-complex in various more stable states (ADP-BeF_3_^−^, ADP-MgF_3_^−^, and AMP-AlF_x_ for ATP ground state, and ADP-AlF_x_ for ATP transition state^27–30^). In the presence of ADP-AlF_x_, the L1s of the PspF_1–275_ hexamer project upward to engage σ^54^ and the co-complex is thought to be in a genuine intermediate state en route to RP_O_ formation.^31,32^ Disruption of the physical linkage between L1 and σ^54^ could potentially lead to an uncoupling phenotype where the ATP hydrolysis energy cannot be relayed. To test whether the activation defects as observed in some of the Cys variants could be attributed to an inability to bind σ^54^, we performed the ADP-AlF_x_ ‘trapping’ assays.

As shown in Table 1, all of the Cys substitutions at the surface-exposed positions in the W56 loop did not exhibit any defect in σ^54^ binding and so must effectively present L1 (in boldface). Amongst the intramolecular Cys variants (Table 1, in italics), the S53C and R55C were sufficient to engage σ^54^ but they were nearly incapable of hydrolysing ATP. This observation suggests that a loss of productive nucleotide hydrolysis rather than an uncoupling phenotype accounts for their RP_O_ formation defects. Variants H50C, F60C, and I61C failed in both σ^54^ binding and ATP hydrolysis. This observation suggests that the two functionally distinct processes are not completely independent and at some level are integrated at the W56 loop.

Taken together, we propose that the W56 loop plays a more significant role in communicating with the ATP hydrolysis site than in organising L1's engagement with σ^54^. This is consistent with the previous observations where upon PspA binding, the ATPase activity of PspF was inhibited at the self-association interface but the σ^54^ binding was not strongly impaired.^23,33^

### W56 loop substitutions promote constitutive higher oligomer formation

**Fig. 3:**
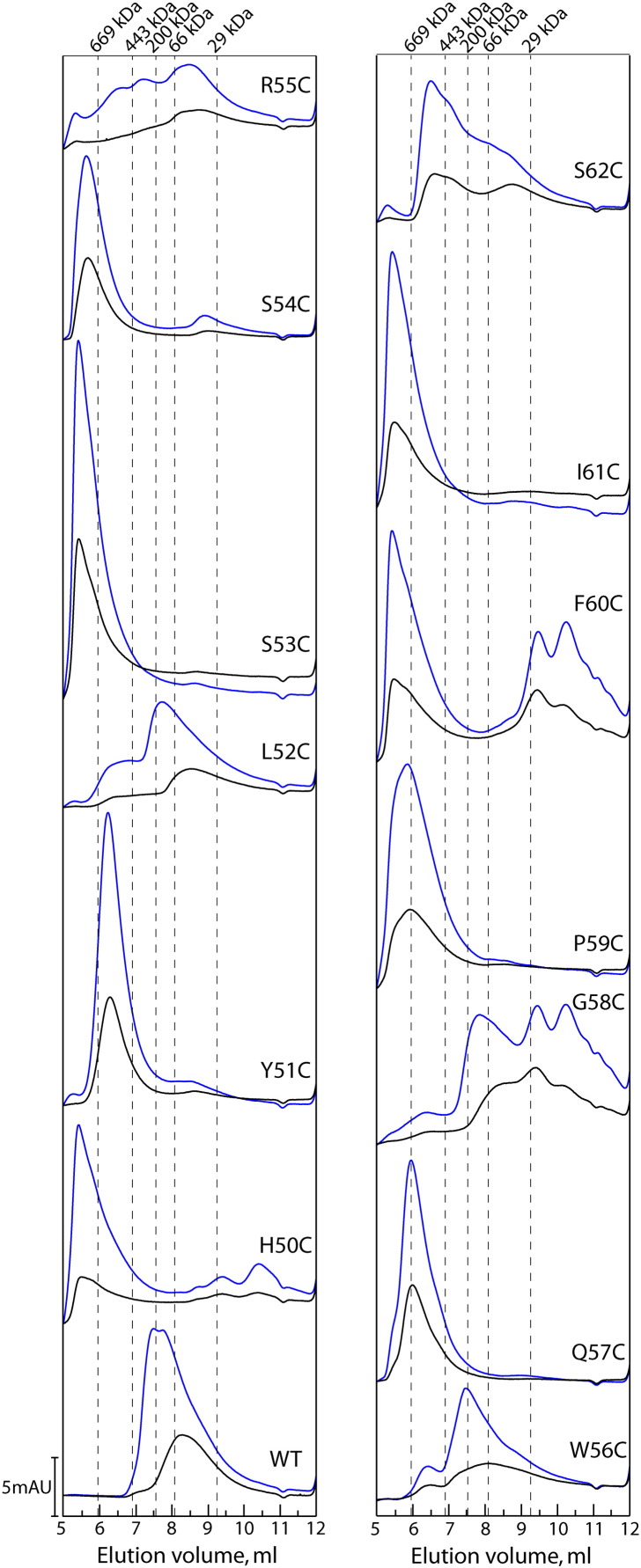
Gel filtration of the W56 loop Cys variants. The experiments were conducted at 4 °C in the absence of any nucleotide using a Yarra Sec-S3000 gel-filtration column (300 × 7.8 mm, Phenomenex). Both high concentration (50 μM, blue trace) and low concentration (20 μM, black trace) of each variant were tested.

To investigate whether the Cys substitutions could affect the overall self-assembly of PspF, we performed gel-filtration chromatography. The oligomeric state of PspF_1–275_ is concentration dependent (e.g., dimers at low injection concentrations and hexamers at higher injection concentrations^34^). Thus, we chromatographed each Cys variant at 20 μM and 50 μM injection concentrations (Fig. 3). Based on individual elution profiles and dominant species, we classified the W56 loop Cys variants into three groups: (i) WT-like apparent hexamers (L52C, W56C, and G58C), (ii) constitutive higher oligomers (typically > 15mers, H50C, Y51C, S53C, S54C, Q57C, P59C, F60C, I61C, and S62C), and (iii) predominantly apparent dimers (R55C). As expected, all the Cys variants that were able to form WT-like apparent hexamers were active in RP_O_ formation (compare Fig. 3 with Fig. 2). The majority of the Cys variants that could form constitutive higher oligomers were either unable or defective in their ability to form RP_O_ (H50C, S53C, P59C, F60C, and I61C, Fig. 2). In contrast, variants Y51C, Q57C, and S62C can activate transcription more efficiently than WT and yet maintain higher-order oligomerisation (Table 1). The higher oligomeric state of the Y51C may explain why it failed to interact with PspA_1–186_. Further studies are required to determine how the interfaces of these higher-order oligomers are organised to generate functional outputs for making RP_O_.

Since the gel-filtration buffer used in the above assays contained no reducing agents, we speculated that the higher-order oligomer formation of the Cys variants could be due to disulfide bonding. We tested two Cys variants (Y51C and S62C) in the presence of 2 mM DTT at 50 μM injection concentrations. They were still able to predominantly form the higher-order oligomers as previously observed (Supplementary Fig 2). We thereby conclude that the higher-order oligomer formation is an intrinsic protein property independent of disulfide bonding.

To summarise, Cys mutations of the W56 loop unexpectedly promote extremely large oligomer formation (commonly 20mers). We propose that the impact of W56 loop mutations on self-association is indirect, because the loop is located at the outer rim of the hexamer and makes only a very limited contact with the quite extensive interface (Fig. 1b and c).

### Both Y51 and W56 are directly involved in PspA_1–186_ binding

**Fig. 4:**
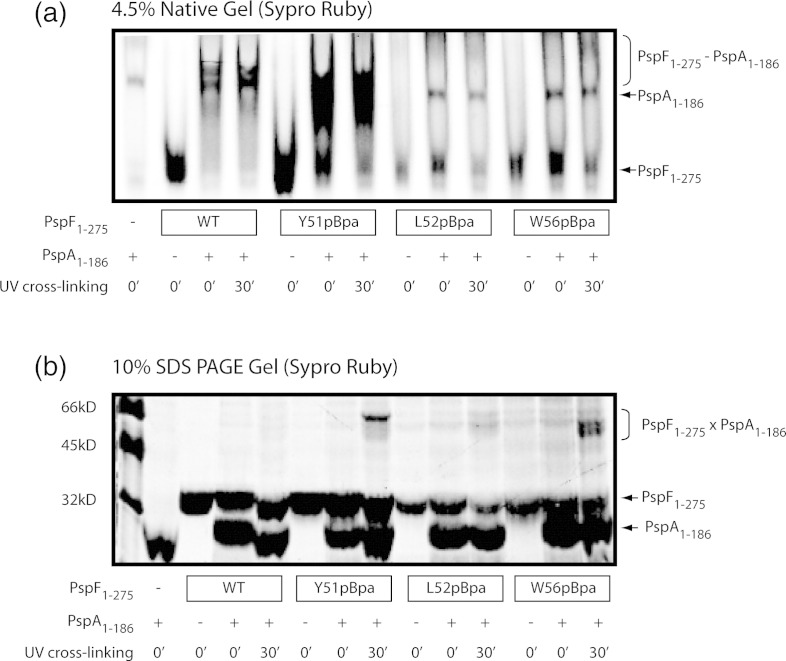
UV cross-linking of the YLW *p*Bpa variants to PspA_1–186_. A photoreactive artificial amino acid *p*Bpa was genetically incorporated to PspF positions Y51, L52, and W56, respectively. The resultant PspF_1–275_ variants Y51*p*Bpa, L52*p*Bpa, and W56*p*Bpa were incubated with PspA_1–186_ and subject to UV irradiation. The samples were loaded on a native gel (a) and on an SDS-PAGE gel (b). The cross-linked species (PspF_1–275_ × PspA_1–186_) are indicated by an open bracket.

In this study, we have identified a key YLW patch in PspF for regulation by PspA. To establish which residue(s) in the YLW patch might be proximal to PspA, we incorporated a photoreactive artificial amino acid *p*Bpa (*p*-benzoyl-l-phenylalanine) into the W56 loop using a *Methanococcus jannaschii* tRNA/tRNA synthetase.^5,35^ The *p*Bpa can cross-link to any C–H bond within 3.1 Å.^35^ The resultant PspF_1–275_ *p*Bpa variants (Y51*p*Bpa, L52*p*Bpa, and W56*p*Bpa) do not seem to generate a significant amount of self-cross-linked products (Supplementary Fig. 3). The Y51*p*Bpa variant was able to bind strongly to PspA_1–186_ (Fig. 4a). When the PspF_1–275_ Y51*p*Bpa–PspA_1–186_ complex was irradiated, a cross-linked species with an apparent molecular mass of 55–60 kDa was observed on the SDS-PAGE gel (Fig. 4b). This cross-linked species corresponds to one PspF_1–275_ Y51*p*Bpa (33 kDa) cross-linked to one PspA_1–186_ (24 kDa). The L52*p*Bpa variant failed to bind PspA_1–186_ and so was unable to yield any cross-linked product (Fig. 4). The W56*p*Bpa variant demonstrated a weak PspA_1–186_ binding ability (consistent with the PspA_1–186_ binding phenotype of the W56C variant, Table 1). Nevertheless, it yielded a significant amount of cross-linked species (Fig. 4b). The W56*p*Bpa × PspA_1–186_ cross-linked species migrates slightly differently from the Y51*p*Bpa × PspA_1–186_ cross-linked species on both native and SDS-PAGE gels (Fig. 4), possibly due to cross-linking to a different site on PspA_1–186_.

In summary, we have provided direct evidence that both Y51 and W56 residues in PspF are directly involved in PspA_1–186_ docking. Given the aromaticity of the bulky *p*Bpa cross-linker (analogous in structure to both Tyr and Trp), we reason that the observed cross-linking events were not due to artificial side-chain extension or local perturbation. Position Y51 is likely to be the primary PspA docking site, whereas position W56 is more likely to be a secondary docking site. Structural data suggest that position L52 points inwards and downwards (Fig. 1b); its role might be predominantly in ATPase regulation (Table 1) rather than directly contributing to PspA docking.

### Multiple PspA binding sites in PspF are needed for repression

**Fig. 5:**
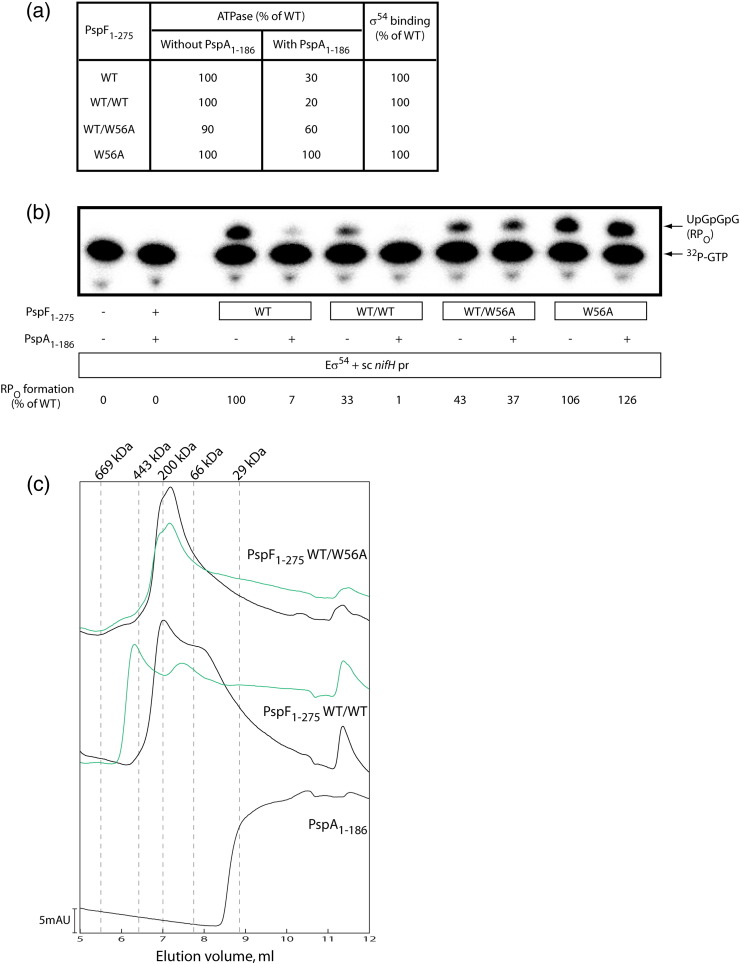
Abolishing three PspA_1–186_ binding sites at alternate positions in a PspF_1–275_ hexamer is sufficient to escape PspA_1–186_ negative regulation. (a) Functional characterisation of the single chain linked PspF_1–275_ dimers (WT/WT and WT/W56A) and the W56A variant. (b) RP_O_ formation assay of the linked PspF_1–275_ dimers and the W56A variant on a supercoiled *nifH* promoter (sc *nifH* pr) in the absence and presence of PspA_1–186_. The amount of RP_O_ with each variant is expressed as a percentage of that of the WT hexamer in the absence of PspA_1–186_. (c) Gel filtration of the linked PspF_1–275_ dimers in the presence and absence of PspA_1–186_. The linked PspF_1–275_ WT/WT and WT/W56A dimers were injected at high concentrations (70 μM) in the absence of any nucleotide at 4 °C using a Yarra Sec-S3000 gel-filtration column (300 × 7.8 mm, Phenomenex). The linked WT/WT and WT/W56A dimers were able to form apparent hexamers (black traces, around 200 kDa). Upon the addition of PspA_1–186_ (green traces), the linked WT/WT hexamers further shifted to apparent dodecamers (around 443 kDa), whereas the linked WT/W56A hexamers showed no significant shift in elution volume.

We previously generated the PspF_1–275_ W56A variant, which is largely refractory to PspA negative regulation.^20^ This variant exhibits WT-like ATPase and σ^54^ binding activities (Fig. 5a); thus, it can serve as an ideal candidate to elucidate the subunit requirement in a PspF_1–275_ hexamer for PspA negative regulation. We constructed single-chain forms of PspF_1–275_ dimers (WT/WT and WT/W56A) by covalently linking two PspF_1–275_ subunits via a Gly-rich sequence.^36^ Both the linked WT/WT and WT/W56A dimers successfully constituted assemblies with apparent molecular weights equivalent to hexamers in gel-filtration chromatography (black traces, Fig. 5c). Functional assays indicate that although the PspF_1–275_ assembly composed of linked WT/WT dimers (the linked WT/WT assembly) does not activate transcription as efficiently as the WT hexamer (approximately 30% of that of the WT hexamer, Fig. 5b), it shows optimal ATPase and σ^54^ binding activities (Fig. 5a^36^). More importantly, the linked WT/WT assemblies are still subject to PspA_1–186_ negative regulation as unlinked subunits (Fig. 5a and b). In the presence of PspA_1–186_, the amount of RP_O_ generated by the linked WT/WT assemblies was reduced by nearly 100%, whereas the amount of RP_O_ generated by the linked WT/W56A assemblies was only reduced by approximately 10% (Fig. 5b). Clearly, the PspF_1–275_ assemblies composed of linked WT/W56A dimers are able to substantially escape PspA_1–186_ negative regulation when only a half of the usual PspA binding sites are operational. To determine the number of PspA_1–186_ subunits bound per linked PspF_1–275_ assembly, we chromatographed the PspA–F complexes through a gel-filtration column (green traces, Fig. 5c). The linked WT/WT assemblies formed apparent dodecamers in the presence of PspA_1–186_, suggesting a stoichiometry of six PspA_1–186_ subunits bound per PspF hexamer (compare green and black traces, Fig. 5c). In contrast, the elution peak corresponding to the linked WT/W56A assemblies did not significantly shift in the presence of PspA_1–186_ (compare green and black traces, Fig. 5c). There might be PspA_1–186_ subunits loosely associated with the linked WT/W56A assembly (recall that only three alternate PspA binding sites are available in a linked WT/W56A assembly), which might easily dissociate from PspF under the gel-filtration conditions. In summary, we propose that in a PspF_1–275_ hexamer, more than three WT subunits, with at least two sharing a self-association interface, are required for PspA_1–186_ to exert full inhibition as a native heteromeric co-complex of six PspA and six PspF subunits.

## Discussion

### W56 loop serves as a primary binding site for PspA

In this study, we have identified a YLW patch within the PspF W56 loop. The primary role of this hydrophobic patch is to serve as a docking site for PspA. We suggest that different roles should now be assigned to individual residues within the YLW patch. Residue Y51 is predominantly responsible for PspA binding, as its *p*Bpa variant strongly cross-links to PspA_1–186_ (Fig. 4b). Residue L52 has a strong functional association with the ATP hydrolytic site (Table 1) and may contribute to the overall hydrophobicity of the PspA binding patch and effects of PspA upon PspF ATPase activity. Residue W56 may serve as a secondary PspA binding site, as its cross-linking pattern to PspA_1–186_ is distinct to that of Y51 (Fig. 4b). Interestingly, the presence of PspA_1–186_ promotes the RP_O_ formation by nearly 20% when residue W56 was mutated to either an Ala (W56A, Fig. 5) or a Cys (W56C, Fig. 2). It would appear that residue W56 plays additional functional roles aside from being just a binding determinant, consistent with a proposed link to the ATPase site of PspF and discussed below in structural terms.^25,36^ Sequence analyses revealed that the YLW patch was not present in all bEBPs. However, its level of hydrophobicity is conserved across different AAA^+^ protein clades (Fig. 1a). Given the importance of the YLW patch in PspA binding and the functional reminiscence of PspA to a *cis*-acting regulatory N-terminal domain of an AAA^+^ protein, we speculate that the YLW patch could have a profound impact on signal transduction from a regulatory domain to an AAA^+^ core.

### PspA binding may uncouple ATPase activity from L1 movement by targeting the W56 loop

**Fig. 6:**
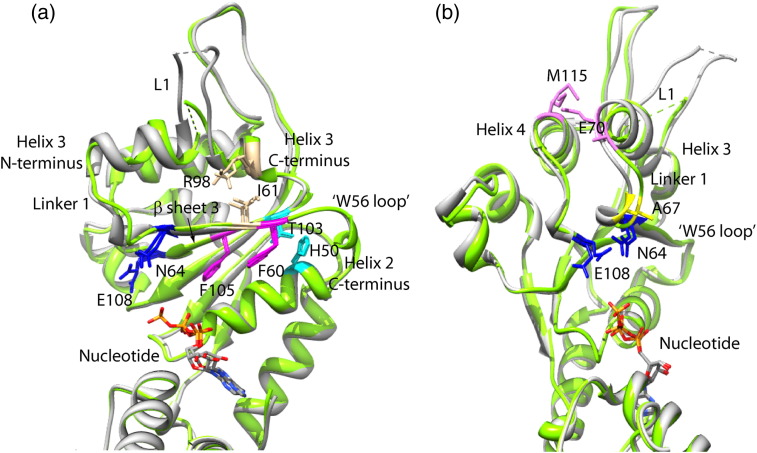
The W56 loop in close association with AAA^+^ domain β-sheet 3/helix 3. The crystal structure of the ATP-bound PspF_1–275_ (PDB entry 2C96, green) and that of the ADP-bound PspF_1–275_ (PDB entry 2C98, grey) were aligned in Chimera. Views parallel to helix 3 (a) and along helix 3 (b) were provided. Interaction pairs were highlighted in the same colour. The glutamate switch residues were also highlighted (N64 and E108 in blue).

Rappas, Nixon *et al.* identified a clear association between PspF L1 movements and the nucleotide occupancy at the hydrolytic site.^32,37^ In the ATP-bound state, the Walker B residue E108 senses the presence of the γ-phosphate and relays this information to the linker 1 residue N64 (in PspF, linker 1 connects the C-terminus of β-sheet 2 to the N-terminus of helix 3, Fig. 6a). Helix 3 changes its orientation and local interaction pairs are disrupted. As a result, L1 assumes an extended conformation to engage σ^54^.^25,32^ In the ADP-bound state, the side chain of residue E108 moves nearly 90° away from N64, so that L1 is locked in a folded state close to helix 3 (Fig. 6b).^25,32^ Residue E108 is highly conserved and serves as a ‘glutamate switch’ for regulating the active state of bEBPs.^38^ We aligned the crystal structures of both ATP- and ADP-bound PspF monomers (green and grey traces, respectively) and noted that the W56 loop made three key interactions to stabilise β-sheet 3 and the C-terminus of helix 3 (Fig. 6a): (i) H50 interacts with T103 via hydrogen bonding, (ii) F60 interacts with F105 via hydrophobic stacking, and (iii) I61 interacts with R98 (a key residue in L1 orientation^25^) via a backbone–side chain interaction. The above interactions provided by the W56 loop appear to fix the C-termini of helices 2 and 3 and β-sheet 3 in space during the ATP hydrolytic cycle (compare the green trace to the grey trace in Fig. 6a).

As a result, outcomes of the hydrolytic events may only manifest as structural changes via linker 1 and the N-terminus of helix 3 to reach L1. Thus, a very important function of the W56 loop is to ensure successfully coupling of nucleotide sensing and L1 projection by defining the undirectionality of the structural changes. Consistent with this proposal, we observed that when the three key W56 loop interactions were disrupted (H50C, F60C, and I61C), L1 lost its ability to engage σ^54^ (Table 1). When the rest of the W56 loop residues were mutated, the ATPase activities were all significantly reduced, whereas the L1 of these variants could still engage σ^54^ (Table 1). This uncoupling phenotype of the W56 loop variants was consistently observed in variants of the glutamate switch pair (Fig. 6, N64–E108 highlighted in blue),^25^ suggesting that the interplay between the two functional motifs is important for PspA to exert its negative regulation on PspF.

We also noted that the PspF linker 1 exhibits the largest conformational movement during the ATP hydrolytic cycle, particularly at residues A67 and E70 (Fig. 6b). Residue A67 is located at the foot of linker 1 and relocates 3.5 Å away from E108 (Fig. 6b). Residue E70 is located at the top of linker 1 and swings nearly 180° towards helix 4 (Fig. 6b). In the ADP-bound state (Fig. 6b grey trace), E70 interacts with residue M115 in helix 4. Interestingly, removal of the side chain of M115 (M115A) increased the initial rate of RP_O_ formation by PspF by nearly 5-fold (Supplementary Fig 4). We speculate that this marked stimulation is attributed to the disruption of the E70–M115 interaction in the ADP-bound state. Thus, PspF L1 is predicted to be projecting upwards to engage σ^54^, somewhat bypassing the folded–extended conformational switches related to nucleotide sensing. Such deregulated L1 movement is interesting because it is an opposite outcome to the uncoupling phenotype of those observed in the W56 loop variants and is rather an increase in coupling. It seems that the coupling reaction is tunable via a number of features of the AAA^+^ core.

### The predicted PspA_1–186_ structure and its relevance to function

**Fig. 7:**
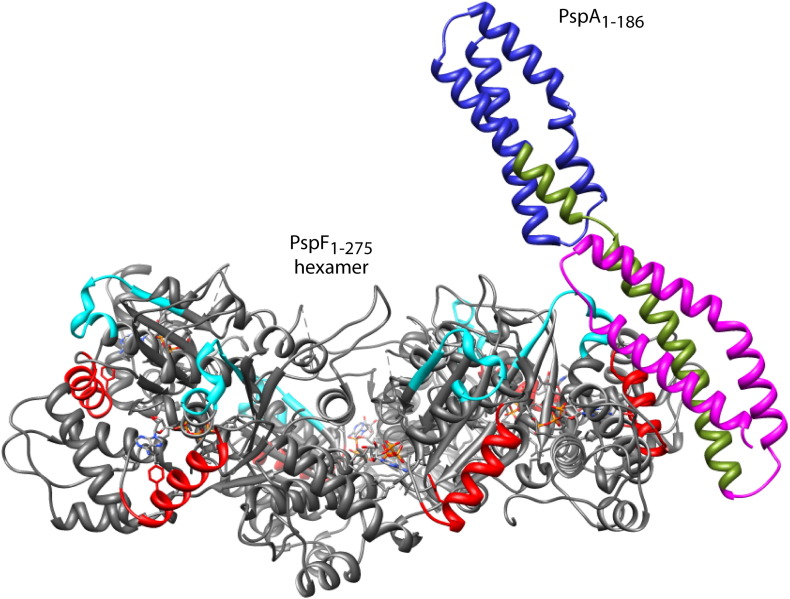
The proposed PspA_1–186_ docking site on PspF. The tertiary structure of PspA_1–186_ was predicted using the I-TASSER server (http://zhanglab.ccmb.med.umich.edu/I-TASSER/), with domains HD1 (residues 1–67, magenta), HD2 (residues 68–110, green), and HD3 (residues 111–186, blue) highlighted. For the purpose of illustration, only one PspA_1–186_ was docked onto the PspF_1–275_ hexamer. We reason that the PspA_1–186_ may function as a *trans*-regulatory domain, reminiscent to the *cis*-regulatory domains in NtrC and NorR, and may be placed at the side of the bEBP hexamer and close to helix α1 (red).^39,40^ As the HD1–2 domains harbour the strongest PspF_1–275_ binding determinants,^23^ they might be interacting with the W56 loop residues (cyan). To not occlude σ^54^ binding,^23^ a domain movement of PspA_1–186_ might occur in a similar fashion as proposed for the regulatory domain of NorR upon activation.^39^

The lack of a PspA structure has been an impediment to the understanding of its docking and inhibition mechanisms. By using the I-TASSER server, we predicted the tertiary structure of the PspA_1–186_ *in silico* and attempted to explain the docking event with PspF based on previous and current biochemical data. The predicted PspA_1–186_ structure has a coiled-coil conformation (Fig. 7), consistent with previous predictions,^20,41^ and shows structural similarity to the alpha spectrin [Protein Data Bank (PDB) entry 1CUN]. The confidence *C*-score for this model, as calculated based on the significance of the threading alignments and the cluster density, is − 2.55. A *C*-score typically ranges from − 5 to + 2, with a higher value reflecting a model of better quality.^42^ The PspA_1–186_ is composed of three domains: HD1 (residues 1–67), HD2 (residues 68–110), and HD3 (residues 111–186). Joly *et al.* observed that HD1 or HD2 alone was not able to interact with PspF or to inhibit its ATPase activity.^23^ HD2–3 only weakly interacts with PspF but has no inhibitory effects.^23^ HD1–2 shows a strong binding affinity towards PspF and inhibits ATP hydrolysis.^23^ From the above observations, we propose that the cooperation of HD1 and HD2 may be essential for PspA docking and inhibition, possibly by directly targeting the W56 loop. If the PspA_1–186_ were to be viewed as a *trans*-acting regulatory domain by functional reminiscence to the classic bEBPs, it could bind near helix α1 of the AAA^+^ core on the side of the bEBP hexameric plane (Fig. 7, red helix). Notably, helix α1 has been inferred as a contact site for the NtrC N-terminal regulatory domain,^40^ and it is located in close proximity to the W56 loop (Fig. 7, compare the cyan loop with the red helix). If correct, a domain reorganisation event would be expected, so that the σ^54^ binding would not be occluded after PspA docking.^23^ We propose that this reorganisation event may take place in a similar fashion as the activated N-terminal regulatory domain of the bEBP NorR.^39^

## Materials and Methods

### Plasmids

Plasmid pPB1 (encoding the *E*. *coli* PspF_1–275_ sequence^26^) was used as a template for site-directed mutagenesis. Each W56 loop position was mutated either to a Cys residue or to an amber stop codon (TAG) for the subsequent artificial amino acid incorporation.

### Computational analyses

The PspF_1–275_ sequence was searched against AAA^+^ proteins using NCBI protein BLAST†. The tertiary structure of PspA_1–186_ was predicted using the I-TASSER online server‡ using the following crystal structures as templates: PDB entries 1CUN, 3S84, 2OTO, 1S35, 2QIH, 3NA7, 3na7A, 1U4Q, 2B5U, and 2YFA. The hexameric PspF_1–275_ structure was previously modelled by M. Rappas with the ATP-bound monomeric PspF_1–275_ crystals (PDB entry 2C96) and subsequently used in two research papers.^5,6^ Protein rendering was performed in Chimera (UCSF).

### Protein expression and purification

The PspF_1–275_ variants were expressed and purified as previously described.^5^ After the (His) × 6 tag cleavage, the PspF_1–275_ variants were stored in the TGED buffer 1 [20 mM Tris–HCl, pH 8.0, 50 mM NaCl, 1 mM DTT, 0.1 mM ethylenediaminetetraacetic acid (EDTA), and 5% glycerol] at − 80 °C. The PspA_1–186_ fragment was expressed, purified, and stored in TGED buffer 2 (20 mM Tris–HCl, pH 8.0, 500 mM NaCl, 1 mM DTT, 0.1 mM EDTA, and 50% glycerol) as previously described.^23^ *Klebsiella pneumoniae* σ^54^ was purified as previously described and stored in TGED buffer 3 (20 mM Tris–HCl, pH 8.0, 200 mM NaCl, 1 mM DTT, 0.1 mM EDTA, and 50% glycerol) at − 80 °C.^5^ *E*. *coli* core RNAP was purchased from Cambio.

### ATPase activity assay

Typically in a 10-μl volume, 4 μM PspF_1–275_ was pre-incubated with the ATPase buffer (20 mM Tris–HCl, pH 8.0, 50 mM NaCl, 15 mM MgCl_2_, 0.1 mM EDTA, and 10 μM DTT) at 37 °C for 5 min. ATP hydrolysis was initiated by addition of 1 mM unlabelled ATP and 0.6 μCi/μl [α-^32^P]ATP (3000 Ci/mmol) and incubated for various time spans at 37 °C. Reactions were quenched by addition of 5 volumes of 2 M formic acid. The [α-^32^P]ADP was separated from the [α-^32^P]ATP by thin-layer chromatography (Macherey-Nagel) in 0.4 M K_2_HPO_4_/0.7 M boric acid. Radioactivity was scanned by PhosphorImager (Fuji Bas-1500) and analysed by Aida software. The ATP turnover rate (*k*_cat_) of each PspF_1–275_ *p*Bpa variant was expressed as a percentage of PspF_1–275_WT activity. All experiments were minimally performed in triplicate.

### Native gel mobility shift assay

The ADP-AlF_x_ trapping reactions were performed in 10 μl volumes and supplemented with 2.35 μM σ^54^, ± 0.3 μM core RNAP, 5 mM NaF, and 4 mM ADP in STA buffer [2.5 mM Tris–acetate, pH 8.0, 8 mM Mg–acetate, 10 mM KCl, 1 mM DTT, 3.5% (w/v) polyethylene glycol 8000] at 37 °C for 5 min. PspF_1–275_ (10 μM) and 0.4 mM AlCl_3_ were added for a further 15 min of incubation to allow ‘trapped’ complex formation at 37 °C. Complexes were analysed on a native gel and stained with SYPRO Ruby stain (Invitrogen).

The PspA–F binding assay was performed in 10 μl volumes supplemented with 10 μM PspF_1–275_ and 30 μM PspA_1–186_ in STA buffer. Complexes were stained with SYPRO Ruby stain, scanned by a Fuji PhosphorImager, and quantified by Aida Image Analyser.

### Gel-filtration assay

Forty-microliter samples were prepared in running buffer (20 mM Tris–HCl, pH 8.0, 50 mM NaCl, and 15 mM MgCl_2_) and centrifuged at 15,000 rpm for 3 min (4 °C) to remove any particulates. The samples were then pipetted into 200-μl crimp autosampler vials. Each sample was placed in the refrigerated autosampler of the Thermo Scientific Surveyor HPLC system. The Yarra Sec-S3000 gel-filtration column (Phenomenex) was attached to the system in a column oven (Phenomenex) at a temperature of 8 °C (the lowest temperature of the oven). The detector was set to detect broad spectrum and UV at 280 nm. The flow rate was set at 1 ml/min for 15 min and the injections were set at 15 μl.

### *p*Bpa-based UV cross-linking assay

The PspA_1–186_-PspF_1–275_ complexes were formed as described above. Reaction mixtures were UV irradiated at 365 nm on ice for 30 min and then analysed on both native and SDS-PAGE gels. The cross-linked protein–protein species were stained by SYPRO Ruby and scanned by a Fuji PhosphorImager.

### *In vitro* RP_O_ formation assay

Open complex formation was measured in 10 μl final volumes containing 4 μM PspF_1–275_, 100 nM holoenzyme (1:4 ratio of E:σ^54^), 20 U RNase inhibitor, 5% (v/v) glycerol, 4 mM dATP, and 20 nM *Sinorhizobium meliloti nifH* promoter in STA buffer at 37 °C. Transcription was activated for various lengths of time before 0.5 mM dinucleotide primer UpG, 0.2 μCi/μl [α-^32^P GTP] (3000 Ci/mmol), and 0.2 mg/ml heparin were added. After extension at 37 °C for 10 min, the reaction mixtures were quenched by addition of 4 μl of 3 × formamide stop dye and resolved on a 20% denaturing sequencing gel. The activator-bypass activities of the σ^54^ variants were examined in a similar experimental procedure without the addition of PspF_1–275_ activators and dATP.

## Author Contributions

N.Z. and M.B. conceived and designed the experiments included in this manuscript. N.Z., T.S., E.L., P.U., N.J., and P.B. performed the experiments.

## Figures and Tables

**Table 1:** Characterisation of the W56 loop Cys variants

PspF_1–275_	RP_O_ formation (% of WT)	ATPase (% of WT)	σ^54^ interaction	PspF_1–186_ binding	Oligomerisation (50 μM concentrations)
WT	100	100	+	+	Hexamer
*H50C*	*0*	*0*	*−*	*+*	*> 20mer*
*S53C*	*25*	*0.2*	*+*	*+*	*> 20mer*
*R55C*	*0*	*0*	*+*	*+*	*Dimer*
*F60C*	*0*	*0.8*	*−*	*+*	*> 20mer*
*I61C*	*0*	*0*	*−*	*+*	*> 20mer*
**Y51C**	**225**	**43**	+	−	> **15mer**
**L52C**	**250**	**17**	+	−	**Hexamer**
**S54C**	**225**	**56**	+	+	> **20mer**
**W56C**	**225**	**57**	+	+	**Hexamer**
**Q57C**	**175**	**8**	+	+	> **20mer**
**G58C**	**375**	**5**	+	+	**Hexamer**
**P59C**	**25**	**0**.**7**	+	+	> **20mer**
**S62C**	**225**	**2**	+	+	> **15mer**

Each Cys variant was tested in its ability to generate RP_O_*in vitro*, to hydrolyse ATP (as a percentage of WT activity), to bind σ^54^ in the presence of ADP-AlF_x_ (an ATP transition-state analogue), to bind PspA_1–186_, and to oligomerise. The W56 loop variants were grouped as the intramolecular variants (H50C, S54C, R55C, F60C, and I61C, in italics) and the surface-exposed variants (Y51C, L52C, S54C, W56C, Q57C, G58C, P59C, and S62C, in boldface).

## Footnotes

† http://blast.ncbi.nlm.nih.gov/

‡ http://zhanglab.ccmb.med.umich.edu/I-TASSER/
