# Supplementary material for: A Key Hydrophobic Patch Identified in an AAA+ Protein Essential for Its In Trans Inhibitory Regulation
Source: J Mol Biol. 2013 Aug 9;425(15):2656–69. doi: 10.1016/j.jmb.2013.04.024 (PMC3791423; doi:10.1016/j.jmb.2013.04.024)
Supplement: Supplementary file 1 — Supplementary materials [file mmc1.pdf]

Supplementary data

Supplementary Fig 1.

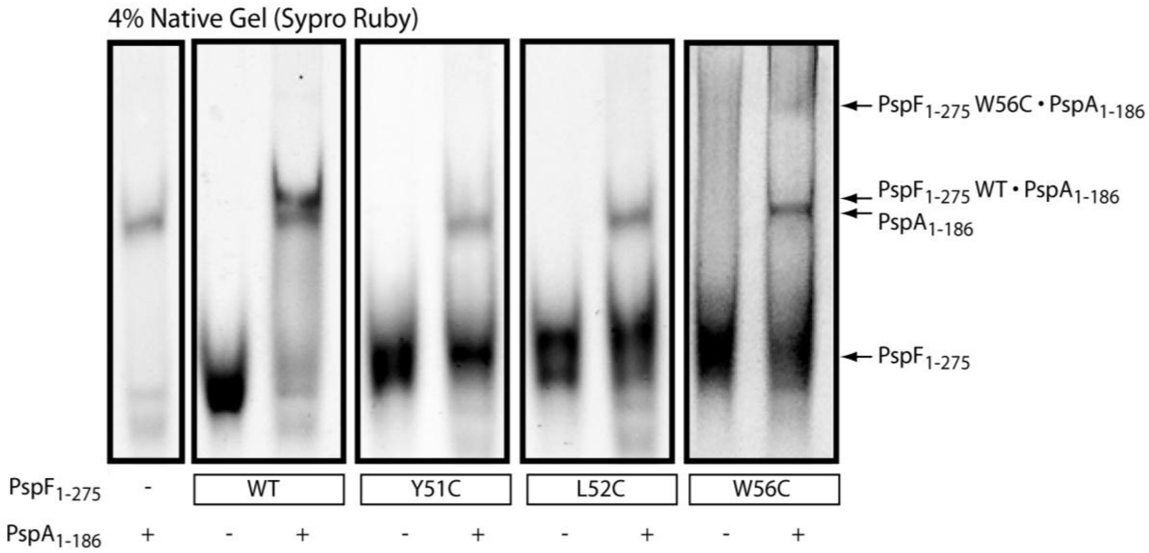

Supplementary Fig 1. Formation of the inhibitory PspA-F complex by $PspF_{1-275}$ 'YLW' Cys variants. Each of the $PspF_{1-275}$ Y51C, L52C and W56C variants was incubated with $PspA_{1-186}$ at 37°C for 15 min and then resolved on a native gel. The Y51C and L52C variants were unable to stably bind $PspA_{1-186}$. The W56C variant was able to form the PspA-F inhibitory complex weakly. However, its migration pattern was significantly different from that of the wildtype PspA-F inhibitory complex.

Supplementary Fig 2.

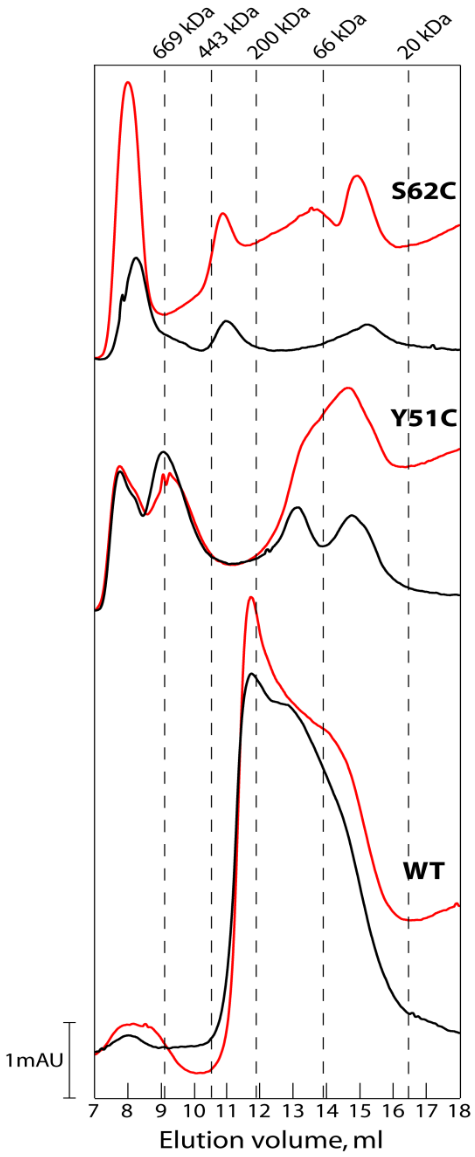

Supplementary Fig 2. Analysing effects of 2 mM DTT on self-association of the Cys variants. The WT and Y51C and S62C variants of PspF at 50 µM injection concentrations were chromatographed through a Superdex 200 column in the absence (black traces) and presence of 2 mM DTT (red traces) respectively. Both Cys variants constitutively formed higher order oligomers in the presence of DTT.

Supplementary Fig 3.

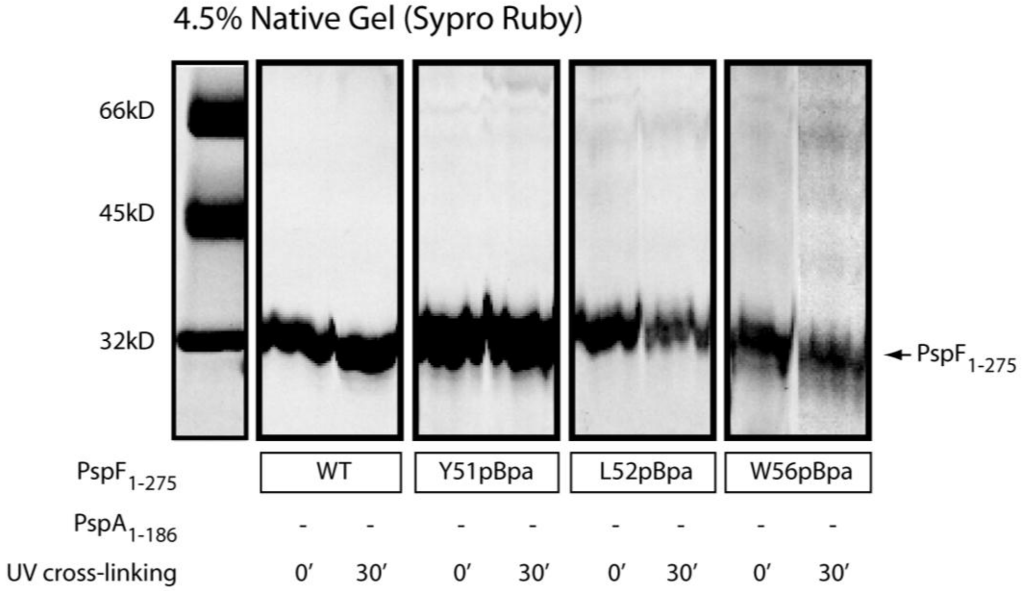

Supplementary Fig 3. Self-crosslinking of the 'YLW' *p*Bpa variants induced by UV irradiation. The Y51*p*Bpa, L52*p*Bpa and W56*p*Bpa variants were subject to either no UV irradiation or 30 min UV irradiation. No significant amount of self-crosslinked species was observed.

Supplementary Fig 4.

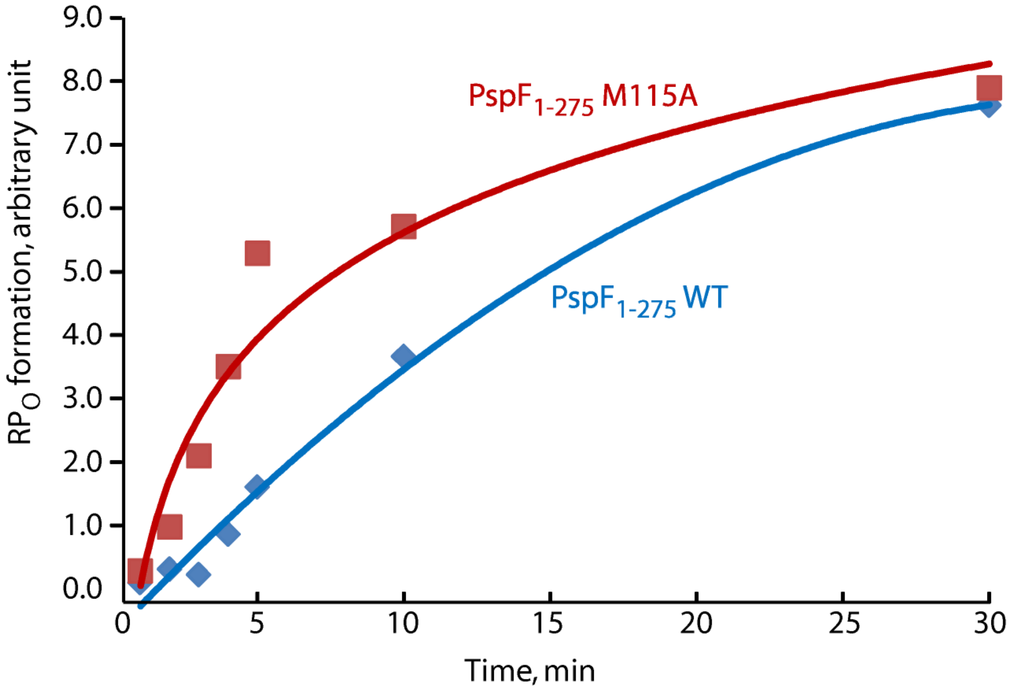

Supplementary Fig 4. RP_O formation assay of $PspF_{1\text{-}275}$ WT and M115A on a supercoiled *S. meliloti nifH* promoter. Each reaction was activated for 1 min, 2 min, 3 min, 4 min, 5 min, 10 min or 30 min before RNA extension was initiated by adding dinucleotide UpG primers and [32P-α] GTP. The amount of RP_O correlates with the amount of transcript UpGpGpG. Variant M115A was nearly 5-fold faster at generating RP_O than WT within the 5 min activation period.
